# Inflammatory panel cytokines are elevated in the neocortex of late-stage Alzheimer’s disease but not Lewy body dementias

**DOI:** 10.1186/s12974-023-02789-8

**Published:** 2023-05-08

**Authors:** Yuek Ling Chai, Jasinda H. Lee, Joyce R. Chong, Clive Ballard, Paul T. Francis, Brian K. Kennedy, Thiruma V. Arumugam, Christopher P. Chen, Dag Aarsland, Mitchell K. P. Lai

**Affiliations:** 1 Department of Pharmacology, Yong Loo Lin School of Medicine, National University of Singapore, Singapore, 117597 Singapore; 2 Memory, Ageing and Cognition Centre, National University Health System, Singapore, Singapore; 3 Department of Biochemistry, Yong Loo Lin School of Medicine, National University of Singapore, Singapore, Singapore; 4 NUS Healthy Longevity Translational Research Programme, Yong Loo Lin School of Medicine, National University of Singapore, Singapore, Singapore; 5 Institute of Psychiatry, Psychology and Neuroscience, King’s College London, London, UK; 6 University of Exeter Medical School, Exeter, UK; 7 Department of Microbiology, Anatomy, Physiology and Pharmacology, School of Agriculture, Biomedicine and Environment, La Trobe University, Bundoora, VIC Australia; 8 Centre for Age-Related Medicine, Stavanger University Hospital, Stavanger, Norway

**Keywords:** Alzheimer’s disease, Cytokines, Dementia with Lewy bodies, Neocortex, Neuroinflammation, Parkinson’s disease dementia

## Abstract

**Background:**

Chronically dysregulated neuroinflammation has been implicated in neurodegenerative dementias, with separate studies reporting increased brain levels of inflammatory mediators and gliosis in Alzheimer’s disease (AD) as well as in Lewy body dementias (LBD). However, it is unclear whether the nature and extent of neuroinflammatory responses in LBD are comparable to those in AD. In this study, we performed head-to-head measurements of a panel of cytokines in the post-mortem neocortex of AD versus the two major clinical subtypes of LBD, namely, dementia with Lewy bodies (DLB) and Parkinson’s disease dementia (PDD).

**Methods:**

Post-mortem tissues from the mid-temporal cortex (Brodmann area 21) of a cohort of neuropathologically well-defined AD, PDD and DLB patients were processed and measured for a comprehensive range of cytokines (IL-1α, IL-1Ra, IL-8, IL-10, IL-12p70, IL-13, IFN-γ, GM-CSF and FGF-2) using a multiplex immunoassay platform. Associations between inflammation markers and neuropathological measures of neuritic plaques, neurofibrillary tangles as well as Lewy bodies were also performed.

**Results:**

We found IL-1α, IFN-γ, GM-CSF and IL-13 to be elevated in the mid-temporal cortex of AD patients. In contrast, none of the measured cytokines were significantly altered in either DLB or PDD. Similar cytokine changes were observed in two other neocortical areas of AD patients. Furthermore, increases of IL-1α, IFN-γ, GM-CSF, IL-10 and IL-13 associated with moderate-to-severe neurofibrillary tangle burden, but not with neuritic plaques or Lewy bodies. Our findings of elevated neocortical pro- and anti-inflammatory cytokines in AD, but not in DLB or PDD, suggest that neuroinflammatory responses are strongly linked to neurofibrillary tangle burden, which is higher in AD compared to LBD. In conclusion, neuroinflammation may not play a prominent role in the pathophysiology of late-stage LBD.

**Supplementary Information:**

The online version contains supplementary material available at 10.1186/s12974-023-02789-8.

## Introduction

Alzheimer’s disease (AD) and Lewy body dementia (LBD) are the two commonest forms of neurodegenerative dementias, both of which are characterised by the loss of cortical neurons thought to be due primarily to abnormal protein aggregation. While neuritic plaques (NP) made up of insoluble, aggregated beta-amyloid (Aβ) peptides and neurofibrillary tangles (NFT) consisting of hyperphosphorylated tau proteins are considered the neuropathological hallmarks of AD, Lewy bodies (LB) arising from aggregation of α-synuclein are a distinct neuropathological feature of LBD. However, we and others [1–3] have reported concomitant but variable burden of NP and NFT in several neocortical regions of LBD patients who did not fulfil clinical or neuropathological diagnostic criteria for AD. This suggests that certain pathophysiological processes which are known to be involved in AD pathogenesis may play a role in LBD as well. For example, chronic glial activation and neuroinflammation have been proposed as important sequelae of amyloidogenesis which may exacerbate tau phosphorylation, neuronal injury and cytotoxicity [4, 5]. Interestingly, tau hyperphosphorylation and subsequent dissociation from cytoskeleton and formation of paired helical filaments have been shown to further promote microglial and astrocyte activation, thus perpetuating a vicious cycle [6, 7].

Given the well-established roles that neuroinflammation play in AD, and the overlapping amyloid and tau pathology between AD and LBD, it is reasonable to postulate the involvement of neuroinflammation in LBD as well. Indeed, a few immunohistochemical and gene expression studies have demonstrated increased cytokine production and markers of microglial as well as astroglial activation [8–10], although others have reported that the extent of gliosis was either not prominent [10–12] or absent [13, 14]. Of the aforementioned studies, relatively few have directly compared AD versus LBD neuroinflammation profiles, although two studies reported lower neuroinflammation markers in LBD compared to AD [9, 13]. Furthermore, potential differences in brain cytokine levels between the two major clinical subtypes of LBD, namely Parkinson’s disease dementia (PDD) and dementia with Lewy bodies (DLB), have not been systematically investigated. Lastly, previous studies have mostly employed immunohistochemical and morphological approaches which, while informative on tissue or cellular localisation of gliotic changes, do not provide quantitative measurements of cortical inflammation mediators. In this study, we aimed to measure a panel of cytokines in specific neocortical areas of subjects with PDD and DLB alongside AD using a quantitative multiplex immunoassay platform. Furthermore, we examined potential links between the measured cytokines and semi-quantitative scores of NP, NFT and LB within our cohort.

## Materials and methods

### Study cohort

All cases were selected based on clinicopathological consensus diagnoses and assessed prospectively by experienced clinicians using validated clinical rating instruments. Clinical classification of LBD was made based on the Movement Disorders Society criteria [15] and the DLB Consortium’s “one-year rule” [16], where PDD was diagnosed when parkinsonism preceded dementia by more than a year while DLB was diagnosed when cognitive impairment or hallucinations were present before or within one year of onset of parkinsonism. AD cases were clinically diagnosed based on the Consortium to Establish a Registry for Alzheimer’s Disease (CERAD) criteria for a diagnosis of probable or definite AD [17]. None of the LBD subjects fulfilled CERAD criteria for concomitant AD. Severity of cognitive impairment was assessed by the annual decline in Mini-Mental State Examination (MMSE) scores from the time of dementia diagnosis to predeath (MMSE decline). Age-matched control cases were also included in this study. Controls were neurologically and cognitively normal, and had only age-associated neuropathological changes and no history of psychiatric diseases. At death, informed consent was sought from next-of-kin before removal of brains, which were collected via University Hospital Stavanger, Norway, and the Brains for Dementia Research network sites, United Kingdom, which include the Thomas Willis Oxford Brain Collections, the London Neurodegenerative Diseases Brain Bank, and Newcastle University. Neuropathological diagnoses were based on Thal Aβ phases [18], neurofibrillary tangle Braak stages [19] and CERAD criteria for AD [17] which are all combined in the National Institute on Aging—Alzheimer’s Association guidelines [20] and the Newcastle/McKeith criteria for Lewy body disease [16]. The collection and study of brain tissues have received Institutional Review Board approval in both the United Kingdom (08/H1010/4) and Singapore (NUS 12-062E) institutions.

### Semi-quantitative pathology scoring

In addition to the above-mentioned neuropathological assessment, semi-quantitative pathology scoring for NP (immunostaining with 4G8 antibody), NFT (immunostaining with AT8 antibody), and α-synuclein-containing LB or neurites (α-synuclein immunostaining) were performed as previously described [3] by neuropathologists blinded to clinical diagnosis on a four point scale: 0 = “None”, 1 = “Sparse”, 2 = “Moderate” and 3 = “Severe”. Each scale was then dichotamised as having non-significant (defined as “None/Sparse”, rating 0–1) versus significant (defined as “Moderate/Severe”, rating 2–3) neuropathological burden for subsequent statistical analyses.

### Brain tissues

Brains were divided into hemispheres after collection, with one hemisphere being formalin fixed for neuropathological assessments, while the other hemisphere was coronally sectioned, dissected to obtain 1-cm^3^ blocks from selected regions, then fresh frozen and stored at − 80 °C. When ready to assay, the frozen brain tissue blocks from the mid-temporal (Brodmann area, BA21), prefrontal (BA9) and parietal (BA40) regions were thawed on ice and dissected free of meninges and white matter, followed by homogenisation using an Ultra-Turrax homogeniser (IKA, Staufen im Breisgau, Germany, on highest setting, 10 s) in ice-cold buffer (50 mM Tris–HCl, 120 mM NaCl, 5 mM KCl, pH 7.4) with cOmplete™ protease inhibitor cocktail and PhosSTOP™ phosphatase inhibitor tablets (Roche Life Science, Penzberg, Germany) at the concentration of 50 mg tissue wet weight/mL.

### Multiplex immunoassays

Immunoassays using the xMAP®-based Luminex platform were performed as previously described [21, 22]. Briefly, tissue homogenates were subjected to agitation on a plate shaker (800 rpm, 40 min) followed by centrifugation at 6000*g* (4 °C, 20 min) to obtain supernatants for inflammatory marker measurements in duplicates using a Luminex assay kit (Catalogue number: HCYTOMAG-60K, Millipore Corp., Billerica, MA, USA) per manufacturer’s instructions. The detectable concentration range of target inflammatory markers is listed in Table 1. Concentration values of each inflammatory marker were normalised with the total protein concentrations measured using Pierce™ Coomassie Plus Reagent (ThermoFisher Scientific, Waltham, MA, USA), and expressed in pg/mg brain protein.

**Table 1:** Detectable concentration range of measured inflammatory markers

Inflammatory marker	Detectable concentration range, Minimum–maximum (in pg/mL)		
	BA21, max *n* = 75	BA9, max *n* = 74	BA40, max *n* = 113
IL-1α	1.0–615.0, *n* = 75	1.9–74.1, *n* = 62	1.5–45.4, *n* = 87
IFN-γ	0.9–277.1, *n* = 75	1.5–61.0, *n* = 69	0.6–41.2, *n* = 113
GM-CSF	0.8–210.0, *n* = 75	1.7–38.5, *n* = 59	0.9–3.6, *n* = 57
IL-13	2.3–205.0, *n* = 75	2.5–155.6, *n* = 74	0.7–59.3, *n* = 112
IL-10	0.3–48.9, *n* = 75	2.2–54.8, *n* = 46	1.7–176.1, *n* = 107
IL-1Ra	1.2–12.1, *n* = 75	n.a	n.a
IL-8	0.3–24.7, *n* = 72	n.a	n.a
IL-12p70	0.8–210.7, *n* = 75	n.a	n.a
FGF-2	935.6–9394.4, *n* = 75	n.a	n.a

BA9: Brodmann area 9 (dorsolateral/medial prefrontal cortex); BA21: Brodmann area 21 (mid-temporal gyrus); BA40: Brodmann area 40 (parietal supramarginal gyrus); FGF-2: Fibroblast growth factor 2; GM-CSF: Granulocyte–macrophage colony-stimulating factor; IFN-γ: Interferon gamma; IL: interleukin; n: number of measurements within detectable range; n.a.: not applicable. Due to limited tissue availability, our study is focused on BA21. Only markers which were significantly altered in BA21 were repeated for BA9 and BA40 to determine whether similar trends apply for different brain regions

### Statistical analyses

Analyses were performed using the SPSS software (version 22, IBM Inc., Armonk, NY, USA). Pearson’s Chi-square tests were used for comparison of gender distribution, while parametric analyses of variance (ANOVA) with Bonferroni post hoc tests were performed to compare normally distributed demographic and clinical variables, namely age at death, post-mortem interval, tissue pH, rate of MMSE decline and duration of dementia among diagnostic groups. Student’s t-tests were used to compare the duration of parkinsonism symptoms between PDD and DLB groups. Given that the cytokine concentrations were not normally distributed (Kolmogorov–Smirnov test, *p* < 0.05), non-parametric Kruskal–Wallis ANOVA with Dunn–Bonferroni post hoc tests were used to compare the concentrations of each cytokine among diagnostic groups, whilst Mann–Whitney U tests were used to compare groups dichotomised according to the severity of neuropathological ratings (None/Sparse vs. Moderate/Severe) for NP, NFT and LB. Effect size estimates for the above-mentioned group comparisons were calculated and expressed in terms of eta-squared (η^2^). Furthermore, logistic regression analyses with covariates adjustment were performed using the R statistical software. To assess the associations between each cytokine and diagnostic groups, multinomial logistic regression analyses were performed with age, gender and duration of dementia included as covariates. Bias-reduction using Firth's penalised maximum likelihood method was done using *brglm2* package, in order to account for the complete separation issue due to duration of dementia. Similarly, binary logistic regression analyses were performed to assess the associations between each cytokine and the presence of Moderate/Severe NFT, with age, gender, NP burden and duration of dementia included as covariates in the regression models. Odds ratios and 95% confidence intervals were computed for all logistic regression models. A *p*-value < 0.05 (two-tailed) and false discovery rate at 10% (i.e. *q*-value < 0.10) was considered statistically significant.

## Results

### Disease and demographic characteristics of study cohort

A total of 77 subjects (18 controls, 22 PDD, 21 DLB and 16 AD) were available for the present study. Table 2 shows that controls and demented patients were well-matched in demographic factors such as age at death, gender distribution, post-mortem interval and tissue pH (as a marker for tissue integrity [23–25]); as well as in the rate of MMSE decline as a marker of dementia severity. While AD patients had the longest duration of dementia among the three dementia groups, DLB patients expectedly had significantly longer mean duration compared to PDD patients. Similarly, there were significant differences in the duration of parkinsonism symptoms, where PDD patients showed longer mean duration than DLB, whilst AD patients had no apparent parkinsonism symptoms. All controls had no to minimal AD pathological changes with Braak stage of 0–II. In agreement with previous observations [3], a higher proportion of DLB patients demonstrated severe Braak stages (V–VI, 42.9%) compared to PDD (4.5%), suggesting more extensive AD-type neuropathological changes in DLB compared to PDD (Table 2). Furthermore, in agreement with previous studies suggesting variable AD pathological burden [1–3], we found that NP and NFT scores were low in PDD (not significantly different from controls) and moderate in DLB. However, both PDD and DLB scores were significantly lower compared to AD (see Table 2).

**Table 2:** Disease and demographic characteristics of study cohort (BA21, max *n* = 75)

	Control	PDD	DLB	AD	*p*-value
Maximum n	18	22	21	14	
Age at death, y	82.8 (1.2)	82.4 (1.0)	83.1 (1.6)	85.6 (1.5)	0.41
Female, %	9 (50.0)	11 (50.0)	11 (52.4)	8 (57.1)	0.98
Post-mortem interval﻿^a^, h	41.5 (5.7)	37.3 (3.6)	44.6 (6.2)	39.0 (6.5)	0.78
Tissue pH	6.4 (0.1)	6.5 (0.1)	6.2 (0.1)	6.4 (0.1)	0.11
MMSE decline per year^b^	NA	2.3 (0.3)	3.0 (0.5)	4.0 (1.2)	0.30
Predeath MMSE^c^	NA	10.9 (8.2)	12.2 (5.1)	8.4 (7.9)	0.52
Duration of dementia^d^, y	NA	3.7 (0.6)	6.4 (0.9)^†^	9.7 (0.8)^†^	< 0.001
Duration of parkinsonism^e^, y	NA	12.9 (1.3)	2.9 (0.7)	NA	< 0.001
Braak stage^f^, n					
0–II	13	15	4	0	NA
III–IV	0	6	8	2	NA
V–VI	0	1	9	11	NA
NP score^g^	0.45 (0.2)	0.63 (0.1)	2.10 (0.2)*^†^	2.92 (0.1)*^†^	< 0.001
NFT score^h^	0.09 (0.1)	0.37 (0.1)	1.40 (0.2)*^†^	2.83 (0.2)*^†‡^	< 0.001
LB scrore^i^	0	0.89 (0.2)*	1.75 (0.3)*	0.25 (0.1)^‡^	< 0.001

All data expressed in mean (SEM) unless otherwise stated. PDD: Parkinson’s disease dementia; DLB: dementia with Lewy body; AD: Alzheimer’s disease; n: number; y: year; h: hour; MMSE: Mini-Mental State Examination; NA: not applicable

^a^Data on post-mortem interval were not available in 1 PDD case

^b^Data on predeath MMSE were not available in all controls, 2 PDD, 9 DLB and 1 AD cases

^c^Data on MMSE decline per year were not available in all controls, 1 PDD, 6 DLB and 2 AD cases

^d^Data on duration of dementia were not available in all controls, 1 PDD, 6 DLB and 1 AD cases

^e^Data on duration of parkinsonism symptoms were not available in all controls, 1 PDD, 7 DLB and 1 AD cases

^f^Data on Braak stage were not available in 5 controls and 1 AD case

^g^Data on NP score were not available in 2 controls, 3 PDD and 1 AD case

^h^Data on NFT score were not available in 2 controls and 1 AD case

^i^Data on LB score were not available in 7 controls, 1 DLB and 2 AD cases

Dunn–Bonferroni post hoc tests correction was performed following a significant Kruskal–Wallis ANOVA), where significant differences from *Control, ^†^PDD and ^‡^DLB were indicated accordingly

### Inflammatory cytokines are elevated in AD but not in LBD

**Fig. 1:**
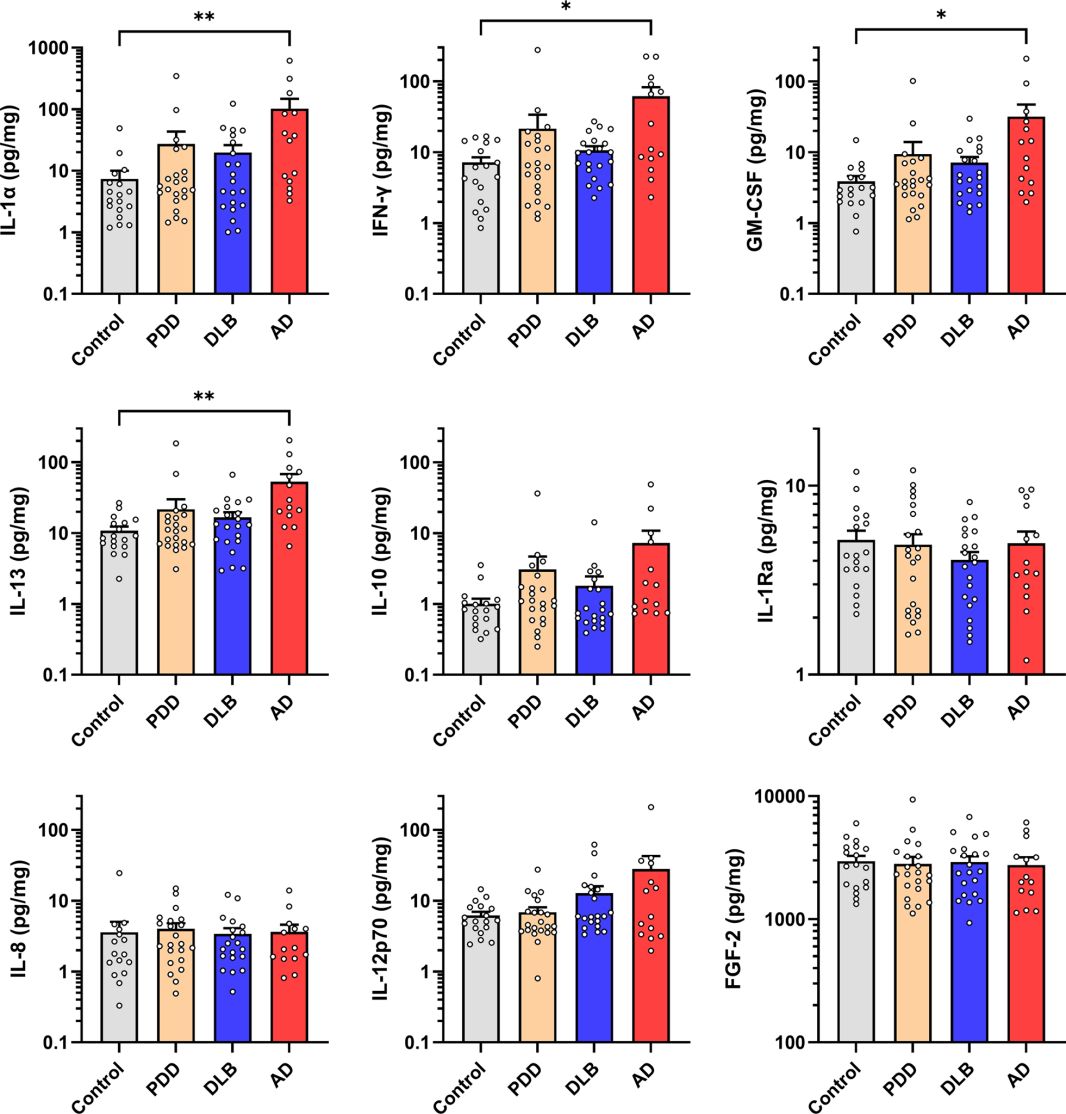
Elevated cytokines in AD temporal cortex. PDD: Parkinson’s disease with dementia; DLB: dementia with Lewy Bodies; AD: Alzheimer’s disease; IL: interleukin; IFN-γ: interferon gamma; GM-CSF: granulocyte-macrophage colony-stimulating factor; FGF-2: fibroblast growth factor 2. Bar graphs of immunoreactivities (in mean ± SEM, with white dots indicating individual measurements) of each inflammatory markers in the temporal cortex as sampled from the mid-temporal gyrus (BA21). *p < 0.05 and **p < 0.01 indicate significant differences between diagnostic groups (Dunn–Bonferroni post hoc tests correction following a significant Kruskal Wallis ANOVA)

A panel of cytokines (IL-1α, IL-1Ra, IL-8, IL-10, IL-12p70, IL-13, IFN-γ, GM-CSF and FGF-2) was initially measured in the tissue homogenates from the mid-temporal gyrus (BA21). Not all assays and measurements were performed for all subjects due to limited tissue availability, and the n numbers of individual experiments are listed in the respective figure legends. Figure 1 shows that IL-1α, IFN-γ, GM-CSF and IL-13 were significantly increased in BA21 of AD patients, but not in either subtype of LBD. A trend towards increased IL-10 was also observed in BA21, but did not reach statistical significance (*p* = 0.062). In order to investigate whether the inflammatory marker changes observed in BA21 may be extended to other neocortical regions, we repeated the measurements of selected cytokines (specifically, those found to be increased in BA21) in the prefrontal (BA9) and parietal (BA40) areas as well. Similar to the changes observed in BA21, none of the inflammatory markers was altered in PDD and DLB (with the exception of increased IFN-γ in DLB), while increased IL-1α was observed in both BA9 and BA40 (Additional file 1: Fig. S1), together with elevated IL-13 and IL-10 in BA40 of the AD subjects (Additional file 1: Fig. S2). The above-mentioned inflammatory cytokines with significant differences among diagnostic groups had medium (η^2^ > 0.06) to large (η^2^ > 0.14) effect sizes, and remained significant after multiple testing correction at False Discovery Rate of 10% (*q* < 0.10). Detailed data descriptions on effect sizes, *p*- and *q*-values are listed in Additional file 1: Table S1. In order to account for the potential confounding effects of demographics factors, multinomial logistic regression analyses with bias-reduction method adjusted for age, gender and duration of dementia were performed to assess the associations between each inflammatory markers in BA21 with diagnostic groups. All five markers in the temporal cortex (i.e. IL-1α, IFN-γ, GM-CSF, IL-13 and IL-10) remained to be significantly associated with AD after adjustment with age and gender (Additional file 1: Table S2, Model I). However, additional adjustment with duration of dementia diminished the statistical significance (Additional file 1: Table S2, Model II).

### Inflammatory cytokines are associated with NFT but not with NP and LB

**Fig. 2:**
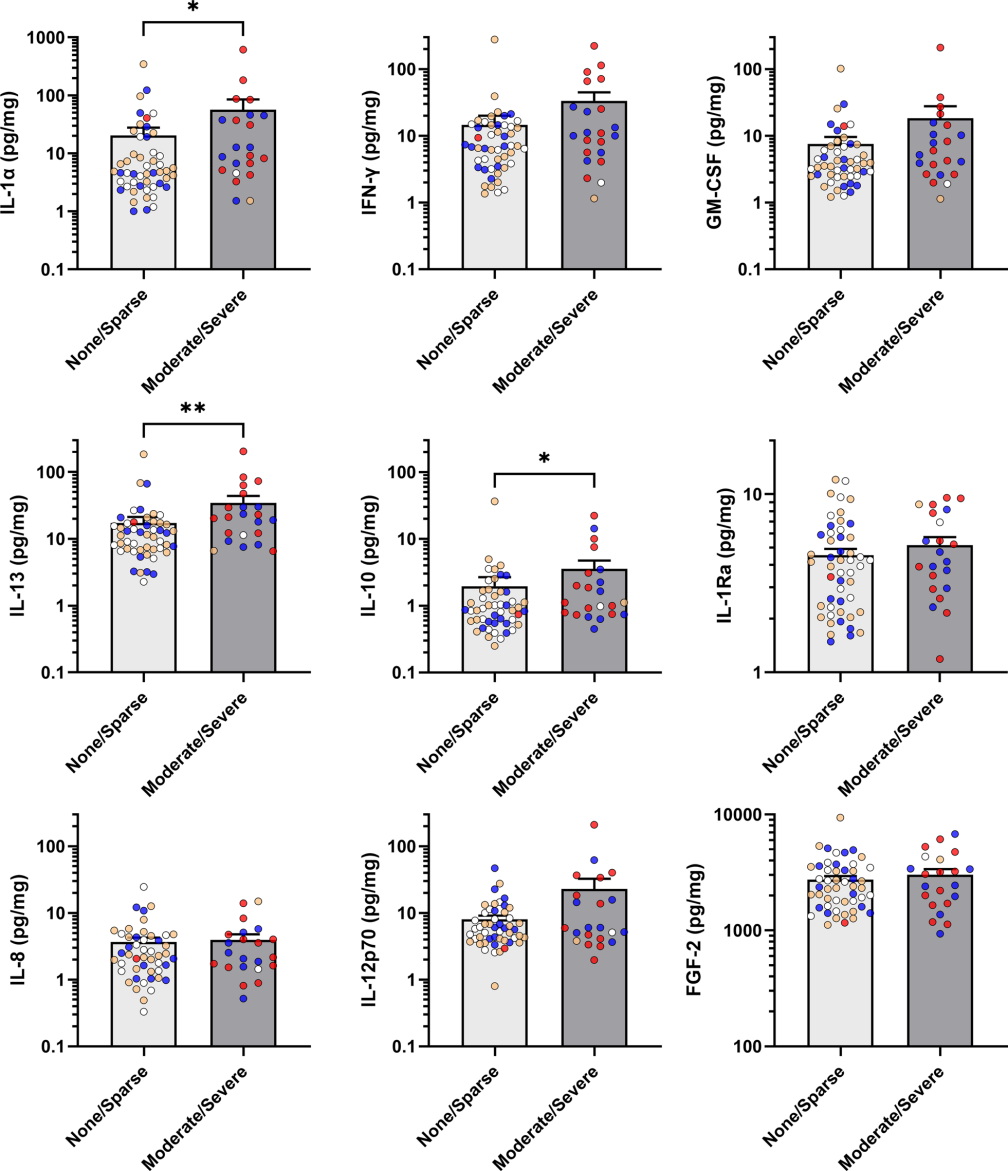
Associations between cytokines and neurofibrillary tangle burden in temporal cortex. NFT: neurofibrillary tangles. Bar graphs of cytokine concentrations (in mean ± SEM pg/mg protein, with coloured dots indicating individual datapoints: white = Control, orange = PDD, blue = DLB, red = AD) of inflammatory markers between None/Sparse versus Moderate/Severe NFT burdens in the temporal cortex as sampled from the mid-temporal gyrus (BA21). *p < 0.05 and **p < 0.01 indicate significant differences between diagnostic groups (Mann–Whitney U tests)

**Fig. 3:**
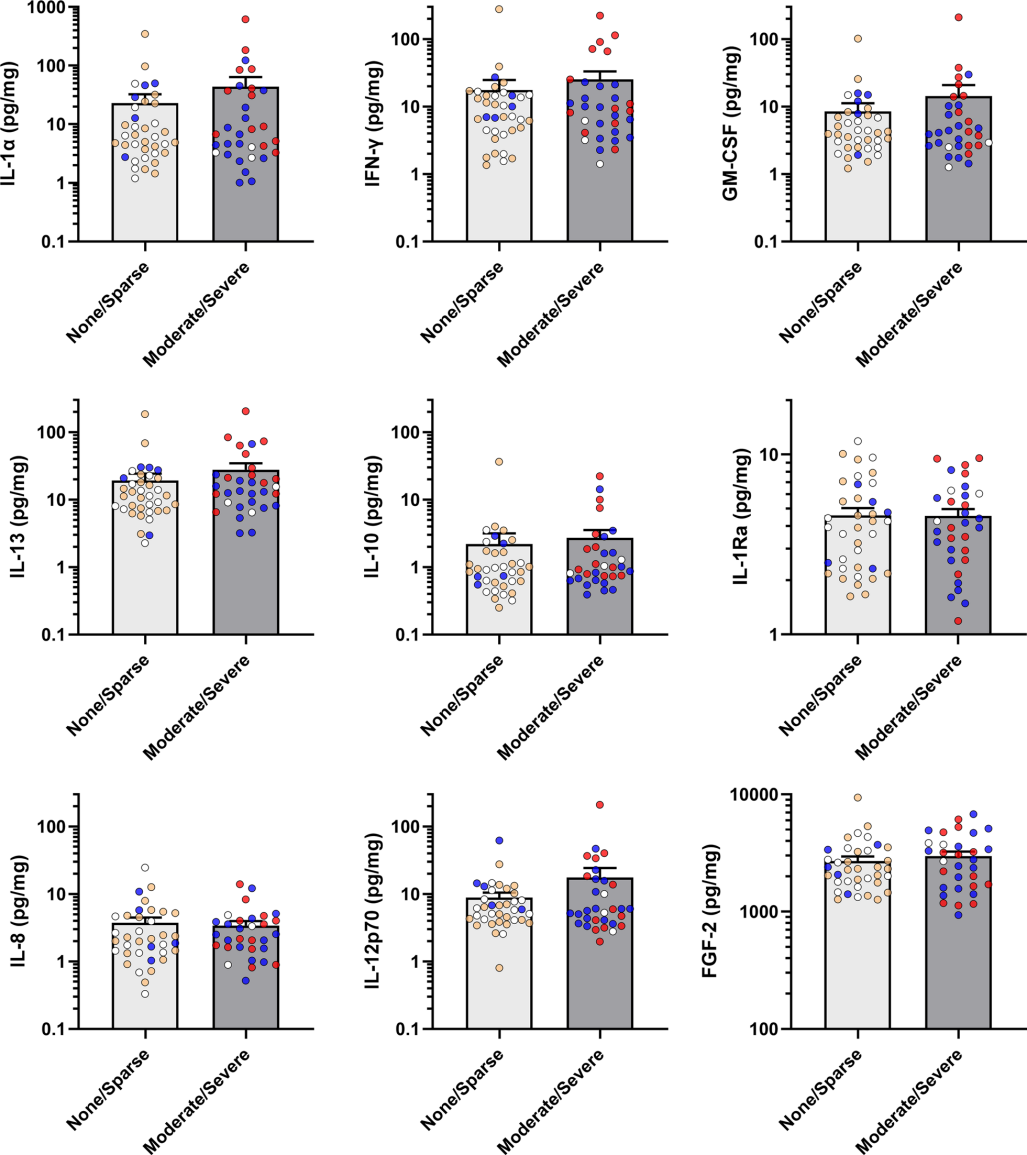
Associations between cytokines and neuritic plaque burden in temporal cortex. NP: neuritic plaques. Bar graphs of cytokine concentrations (in mean ± SEM pg/mg protein, with coloured dots indicating individual datapoints: white = Control, orange = PDD, blue = DLB, red = AD) of inflammatory markers between None/Sparse versus Moderate/Severe NP burden groups in the temporal cortex as sampled from the mid-temporal gyrus (BA21)

**Fig. 4:**
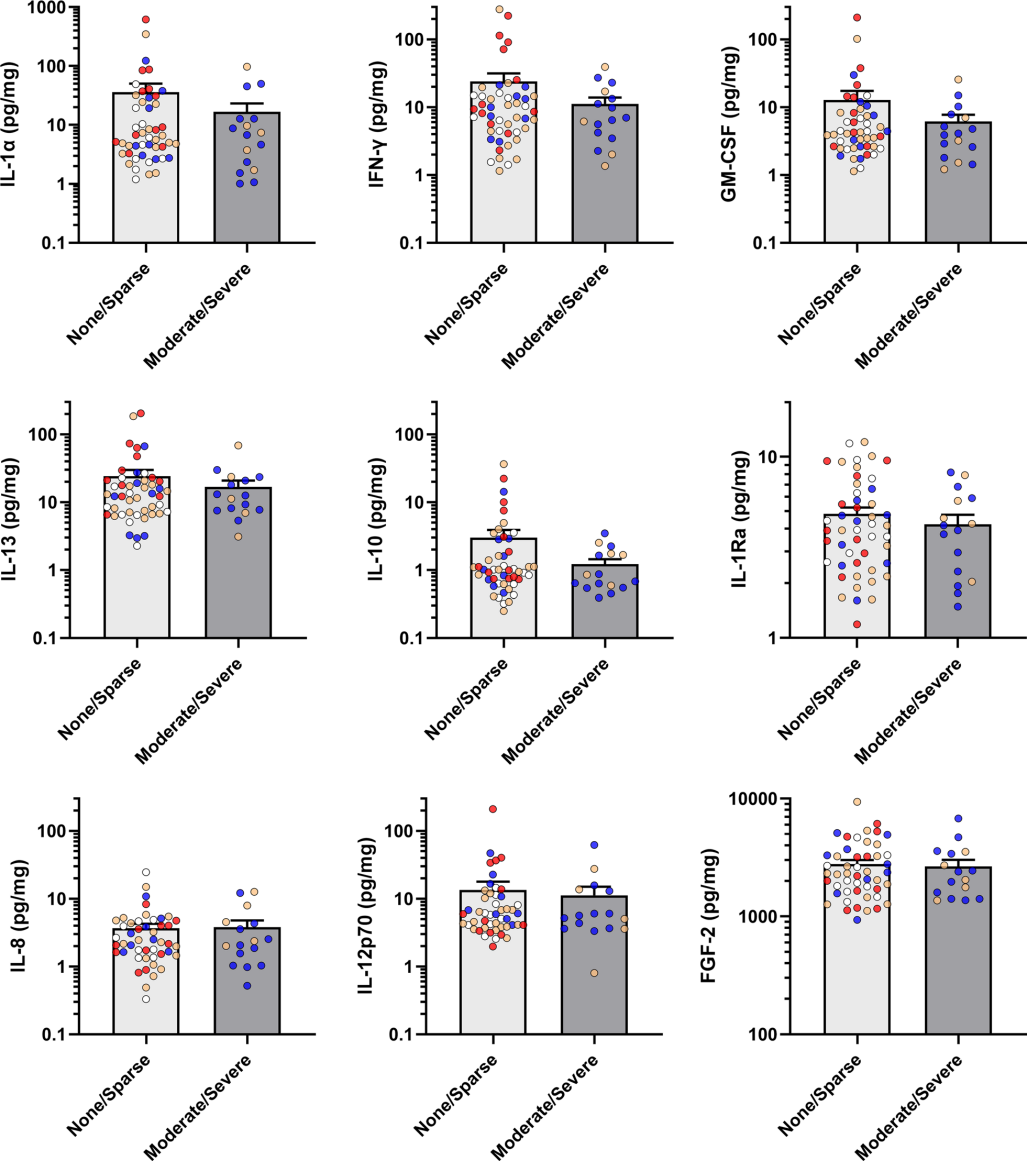
Associations between cytokines and Lewy body burden in temporal cortex. LB: Lewy bodies. Bar graphs Bar graphs of cytokine concentrations (in mean ± SEM pg/mg protein, with coloured dots indicating individual datapoints: white = Control, orange = PDD, blue = DLB, red = AD) of inflammatory markers between None/Sparse versus Moderate/Severe NP burden groups in the temporal cortex as sampled from the mid-temporal gyrus (BA21)

Figure 2 shows that most inflammatory cytokines which are elevated in BA21 of AD subjects were found to be elevated in the presence of significant (“Moderate/Severe” rating) NFT burden. These include both pro-inflammatory (IL-1α) and anti-inflammatory (IL-10 and IL-13) cytokines. All significantly elevated cytokines had medium effect size (η^2^ > 0.06), with IL-1α and IL-13 retaining statistical significance at FDR = 10% (*q* < 0.10). In contrast, none of the inflammatory cytokines was associated with “Moderate/Severe” NP (Fig. 3**)** or LB (Fig. 4) ratings. Detailed data descriptions on effect sizes, *p*- and *q*-values are listed in Additional file 1: Table S3. In order to account for the potential confounding effects of other factors, binary logistic regression analyses adjusted for age, gender, presence of Moderate/Severe NP and duration of dementia were performed to examine the associations between each inflammatory marker and NFT. IL-1α, IFN-γ, GM-CSF and IL-13 were found to be significantly associated with Moderate/Severe NFT, after adjustment with age, gender and NP (Additional file 1: Table S4, Model I). However, additional adjustment with duration of dementia diminished the statistical significance (Additional file 1: Table S4, Model II).

## Discussion

PDD and DLB share overlapping pathophysiology with AD, and multiple studies have suggested the inclusion of reactive glial cells and chronic neuroinflammation as major contributors to the pathogenesis and progression of all three neurodegenerative dementias. However, whilst inflammatory activation is a well-established finding in the AD brain, post-mortem studies on the status of inflammatory mediators in PDD and DLB have been inconsistent (see Introduction). Using a sensitive multiplexing platform, we performed head-to-head quantitative comparisons of a panel of inflammatory cytokines in PDD, DLB as well as AD neocortex. We confirmed a robust neuroinflammatory activation in AD as indicated by elevations of several cytokines in the temporal cortex, with similar observations for the prefrontal and parietal lobes. In contrast, brain cytokines levels were not significantly changed in PDD and DLB. Our data suggest that, in contrast to AD, neuroinflammatory activation is likely limited in both severity and extent in LBD neocortex [9, 12–14].

LBD, although characterised primarily by LB pathology, displayed variable degrees of AD pathological features (i.e. NP and NFT) [1–3]. In agreement with those studies, we found low-to-moderate NFT scores in both PDD and DLB, which were significantly lower compared to AD (see Table 1). Interestingly, the current results showed that brain cytokines were significantly elevated only in the presence of moderate-to-severe NFT burden, whilst not directly associated with NP or LB. Therefore, we postulate that the lack of elevated inflammatory markers in LBD neocortex may be related to the relatively lower NFT burden. Furthermore, the lack of significant differences in brain cytokines between PDD and DLB may be explained by the smaller variations in NFT scores between these two LBD subtypes relative to AD. Although our findings of brain cytokines associations with NFT but not NP seem to contradict the reported immunogenicity of Aβ peptides during AD pathogenesis [4, 5], it is worth noting that the supporting data for those studies were derived from in vitro and animal-based studies utilising oligomeric forms of Aβ, rather than the highly aggregated, beta-pleated sheet form typically found in NP [26]. Indeed, plaque-residing Aβ may be the product of microglial action which served to sequester excess levels of the peptide, hence limiting its neurotoxicity [27, 28]. Therefore, it is possible that plaque-residing Aβ may have different properties, including immunogenicity, compared to oligomeric species. With regard to LBs whose main component, α-synuclein fibrils, do not appear to have significant immunogenicity [29] or to correlate neatly with clinical decline, especially in PDD [30], our finding of a lack of associations between LB scores and cytokine markers suggest that these markers are driven primarily by NFT, and the low levels of cytokines found in PDD and DLB may thus reflect relatively low NFT burden in Lewy body dementias (see Table 1, [3]). Importantly, the current data are congruent with the postulated bi-directional links between neuroinflammation and NFT formation. For example, while inflammatory cytokines may induce tau hyperphosphorylation and NFT formation, hyperphosphorylated tau is also immunogenic and triggers glial activation, thus forming a vicious cycle that drives disease progression, leading to neurodegeneration and cognitive impairment [6, 7]. In terms of AD pathological features, it has become increasingly clear that NFT is a superior predictor of neuropathological and clinical outcomes such as brain atrophy and cognitive decline compared to Aβ [31–34]. The extent that neuroinflammation constitutes an important pathophysiological mechanism for neurodegenerative processes thus provides one explanation underlying the more proximal associations observed between inflammatory cytokines and NFT.

In considering the observed differences of neuroinflammatory responses between dementia types, one important caveat is the potential effects of dementia duration, which is by definition shorter in PDD compared to DLB and AD [16], and shows covariate effects both with AD (see Additional file 1: Table S2) as well as with NFT burden (see Additional file 1: Table S4) associations with inflammatory markers. This link may be bidirectional, for example, where the longer dementia duration of AD facilitates greater accumulation of immunogenic NFT, in turn leading to higher inflammatory markers. On the other hand, the early presence of chronic neuroinflammation may predispose formation of NFT and other neuropathological markers, in turn associating with earlier, more severe onset of cognitive decline. Further work is needed to delineate the complex interactions between neuroinflammation, disease-specific processes and clinical factors such as dementia duration.

This study has several limitations. Firstly, while our study investigated a range of cytokines, we did not cover a full spectrum of inflammatory mediators and signalling molecules pathways, and it is possible that pathway analytes not measured at present may be significantly activated in LBD. Future “omics”-based studies should be performed to gain a more complete understanding of alterations of the neuroinflammation network in neurodegenerative dementias. For instance, publicly deposited databases from the Religious Orders Study/Memory and Aging Project (ROSMAP) may be particularly resourceful. Several groups had profiled brain inflammatory markers in non-demented and AD subject in the ROSMAP cohorts [35, 36]; this could be expanded into studying LBD and serve as an important validation of our findings. Secondly, although we analysed three separate neocortical regions showing broadly similar trends of inflammatory marker alterations, the highest number of measurements were focused at the mid-temporal cortex (BA21), which has previously been shown to be differentially affected by AD versus LBD processes [37]. Therefore, it is possible that brain regions not currently sampled may manifest higher levels of neuroinflammatory activation in LBD. Follow-up work which measures brain inflammatory markers in multiple cortical and subcortical regions will provide further insights into the patterns of neuroinflammatory responses as well as their relationship with neuropathological markers in AD and LBD. Finally, whilst our data indicate robust associations between inflammatory cytokines and NFT, the underlying mechanisms and causal relationship between inflammation and tangle-related pathology could be speculated on (see above), but not confirmed at present. More importantly, given that LBD cases often display variable NFT pathology, validation studies in a separate LBD cohort with a wider range of NFT burden is needed to better define the potential effect of higher NFT load on neuroinflammation in LBD.

In conclusion, using an extensive panel of Luminex-based inflammatory markers measurement on post-mortem neocortical tissues from a well-characterised patient cohort, this study reported elevations of several cytokines in the neocortex of AD which were associated with NFT severity. On the other hand, both DLB and PDD did not show significant cytokine changes, suggesting that in contrast with AD, neuroinflammatory activation may not play a prominent role in the pathophysiology of LBD. One implication of the current results is that biomarkers and therapeutic strategies targeting neuroinflammation may have less utility in LBD compared to AD.

## Supplementary Information

**Additional file 1: Table S1**. Multiple comparisons of each inflammatory marker among diagnostic groups, using non-parametric Kruskal–Wallis ANOVA tests.** Table S2**. Associations between each inflammatory marker in temporal cortex with diagnostic groups.** Table S3**. Multiple comparisons of each inflammatory marker in the presence versus absence of neuropathological features, using non-parametric Mann–Whitney U tests.** Table S4**. Associations between each inflammatory marker in temporal cortex with the presence of Moderate/Severe NFT.** Figure S1**. Pro-inflammatory markers in frontal and parietal lobes of AD and LBD.** Figure S2**. Anti-inflammatory markers in frontal and parietal lobes of AD and LBD.

## Data Availability

Anonymised datasets analysed in this study are available from the corresponding author on reasonable request.

## Abbreviations

AD: Alzheimer’s disease
ANOVA: Analyses of variance
Aβ: Beta-amyloid
BA: Brodmann area
CERAD: Consortium to Establish a Registry for Alzheimer’s Disease
DLB: Dementia with Lewy bodies
FGF-2: Fibroblast growth factor 2
GM-CSF: Granulocyte-macrophage colony-stimulating factor
IFN-γ: Interferon gamma
IL: Interleukin
LB: Lewy bodies
LBD: Lewy body dementia
MMSE: Mini-Mental State Examination
NFT: Neurofibrillary tangles
NP: Neuritic plaques
PDD: Parkinson’s disease dementia
