# Supplementary material for: Inflammatory panel cytokines are elevated in the neocortex of late-stage Alzheimer’s disease but not Lewy body dementias
Source: J Neuroinflammation. 2023 May 8;20:111. doi: 10.1186/s12974-023-02789-8 (PMC10169342; doi:10.1186/s12974-023-02789-8)
Supplement: Supplementary file 1 — Additional file 1: Table S1. Multiple comparisons of each inflammatory marker among diagnostic groups, using non-parametric Kruskal–Wallis ANOVA tests. Table S2. Associations between each inflammatory marker in temporal cortex with diagnostic groups. Table S3. Multiple comparisons of each inflammatory marker in the presence versus absence of neuropathological features, using non-parametric Mann–Whitney U tests. Table S4. Associations between each inflammatory marker in temporal cortex with the presence of Moderate/Severe NFT. Figure S1. Pro-inflammatory markers in frontal and parietal lobes of AD and LBD. Figure S2. Anti-inflammatory markers in frontal and parietal lobes of AD and LBD. [file 12974_2023_2789_MOESM1_ESM.pdf]

Chai *et al*. Inflammatory markers are elevated in the neocortex of Alzheimer's Disease but not Lewy Body Dementias

Additional file

Table S1. Multiple comparisons of each inflammatory marker among diagnostic groups, using non-parametric Kruskal-Wallis ANOVA tests.

		IL-1α	IFN-γ	GM-CSF	IL-13	IL-10	IL-1Ra	IL-8	IL-12p70	FGF-2
BA 21	n	75	75	75	75	75	75	72	75	75
	H	12.340	8.994	10.090	14.650	7.349	1.646	1.694	4.154	1.122
	η2	0.132	0.084	0.100	0.164	0.061	-0.019	-0.019	0.016	-0.026
	p-value	**0.006**	**0.029**	**0.018**	**0.002**	0.062	0.649	0.638	0.245	0.772
	q-value	**0.028**	**0.066**	**0.053**	**0.019**	0.111	0.730	0.730	0.368	0.772
BA 9	n	62	69	59	74	46	n.a.	n.a.	n.a.	n.a.
	H	12.820	2.564	6.903	6.841	5.087				
	η2	0.169	-0.007	0.071	0.055	0.050				
	p-value	**0.005**	0.464	0.075	0.077	0.166				
	q-value	**0.025**	0.464	0.129	0.129	0.207				
BA 40	n	87	113	57	112	107	n.a.	n.a.	n.a.	n.a.
	H	9.271	9.529	7.431	10.510	14.170				
	η2	0.076	0.060	0.084	0.070	0.108				
	p-value	**0.026**	**0.023**	0.059	**0.015**	**0.003**				
	q-value	**0.032**	**0.032**	**0.059**	**0.032**	**0.014**				

Abbreviations: BA = Brodmann area, n = number of measurements, H = Kruskal-Wallis H-statistic, η^2 = eta2 squared based on H-statistic: (H - k + 1)/(n - k) where k = 4 diagnostic groups, n.a. = not applicable.

Bold fonts indicate significant differences between diagnostic groups, $p < 0.05$ (Kruskal-Wallis test) and $q < 0.10$ (False Discovery Rate of 10%).

Table S2. Associations between each inflammatory marker in temporal cortex with diagnostic groups.

BA21 marker		Odds Ratio (95% Confidence Interval)		
		PDD	DLB	AD
IL1-α	Model I	2.51 (0.68, 9.22)	2.76 (0.75, 10.22)	**8.36 (2.01, 34.78)**
	Model II	1 (0.09, 11.35)	0.91 (0.06, 12.87)	1.89 (0.12, 30.05)
IFN-γ	Model I	2.32 (0.53, 10.15)	3.09 (0.7, 13.67)	**11.7 (2.17, 63.12)**
	Model II	0.72 (0.06, 8.2)	1.27 (0.08, 19.08)	3.15 (0.17, 58.38)
GM-CSF	Model I	3.17 (0.49, 20.68)	4.55 (0.7, 29.67)	**16.56 (2.22, 123.71)**
	Model II	0.76 (0.03, 17.56)	1.05 (0.03, 32.3)	2.29 (0.06, 83.16)
IL-13	Model I	3.07 (0.42, 22.47)	3.15 (0.42, 23.35)	**38.78 (3.78, 397.88)**
	Model II	0.81 (0.02, 29.83)	0.54 (0.01, 30.66)	4.6 (0.06, 329.71)
IL-10	Model I	4.1 (0.61, 27.41)	2.5 (0.35, 17.7)	**10.2 (1.36, 76.3)**
	Model II	3.02 (0.1, 95.59)	0.86 (0.02, 47.75)	3.47 (0.06, 195.12)
IL-1ra	Model I	0.37 (0.02, 5.91)	0.14 (0.01, 2.41)	0.37 (0.02, 8.66)
	Model II	0.25 (0, 182.88)	0.08 (0, 92.49)	0.18 (0, 300.5)
IL-8	Model I	2.38 (0.41, 13.84)	1.8 (0.29, 10.95)	2.23 (0.3, 16.63)
	Model II	0.67 (0.01, 30.39)	0.2 (0, 12.68)	0.12 (0, 11.18)
IL-12p70	Model I	0.87 (0.11, 7.12)	4.44 (0.61, 32.3)	6.04 (0.73, 49.8)
	Model II	0.9 (0, 924)	1.83 (0, 2328.01)	1.97 (0, 2706.73)
FGF-2	Model I	0.42 (0.02, 7.61)	0.64 (0.04, 11.7)	0.32 (0.01, 8.34)
	Model II	1.67 (0, 926.9)	3.49 (0, 3561.64)	1.12 (0, 1983.26)

Abbreviations: PDD = Parkinson's disease with dementia, DLB = dementia with Lewy Bodies, AD = Alzheimer's disease. Inflammatory markers were log-10 transformed. Model I was adjusted for age and gender, while Model II was adjusted for age, gender and duration of dementia.

Bold fonts indicate significant associations between inflammatory marker and diagnostic group (p<0.05, multiple multinomial regression analysis with bias-reduction using Firth's penalized maximum likelihood method).

Table S3. Multiple comparisons of each inflammatory marker in the presence versus absence of neuropathological features, using non-parametric Mann-Whitney U tests.

		IL-1α	IFN-γ	GM-CSF	IL-13	IL-10	IL-1Ra	IL-8	IL-12p70	FGF-2
NFT	n	72	72	72	72	72	72	70	72	72
	Z	2.482	1.687	1.797	2.983	2.066	1.076	0.392	1.167	0.587
	η2	0.087	0.040	0.045	0.125	0.060	0.016	0.002	0.019	0.005
	p-value	**0.013**	0.092	0.072	**0.003**	**0.039**	0.282	0.695	0.243	0.557
	q-value	**0.059**	0.165	0.163	**0.026**	0.116	0.363	0.695	0.363	0.627
NP	n	69	69	69	69	69	69	67	69	69
	Z	0.505	0.397	0.349	1.300	0.908	0.090	0.289	0.349	0.686
	η2	0.004	0.002	0.002	0.025	0.012	0.000	0.001	0.002	0.007
	p-value	0.613	0.691	0.727	0.194	0.364	0.928	0.772	0.727	0.493
	q-value	0.869	0.869	0.869	0.869	0.869	0.928	0.869	0.869	0.869
LB	n	65	65	65	65	65	65	63	65	65
	Z	-0.510	-0.472	-0.579	-0.015	-0.899	-0.503	-0.095	-0.122	-0.091
	η2	0.004	0.003	0.005	0.000	0.013	0.004	0.000	0.000	0.000
	p-value	0.610	0.637	0.563	0.988	0.369	0.615	0.925	0.903	0.927
	q-value	0.988	0.988	0.988	0.988	0.988	0.988	0.988	0.988	0.988

Abbreviations: NFT = neurofibrillary tangles, NP, neuritic plaques, LB = Lewy bodies, Z = Z-score from Mann-Whitney U test, η^2 = eta2 squared based on Z-statistic: $Z^2/(n - 1)$.

Bold fonts indicate significant differences between diagnostic groups, $p < 0.05$ (Kruskal-Wallis test) and $q < 0.10$ (False Discovery Rate of 10%).

Table S4. Associations between each inflammatory marker in temporal cortex with the presence of Moderate/Severe NFT.

Log-10 transformed marker (BA21)	Presence of Moderate/Severe NFT Odds ratio (95% Confidence Interval), p-value	
	Model I (max n = 21)	Model II (max n = 18)
IL-1α	**3.69 (1.25, 10.9), *p* = 0.018**	3.55 (0.95, 13.22), *p* = 0.059
IFN-γ	**4.04 (1.04, 15.6), *p* = 0.043**	3.18 (0.67, 15.01), *p* = 0.144
GM-CSF	**4.87 (1.07, 22.22), *p* = 0.041**	3.98 (0.65, 24.18), *p* = 0.134
IL-13	**12.73 (1.84, 88.01), *p* = 0.010**	**25.36 (1.94, 332.24), *p* = 0.014**
IL-10	4.68 (0.98, 22.43), *p* = 0.054	4.39 (0.58, 33.35), *p* = 0.153
IL-1Ra	6.72 (0.34, 131.96), *p* = 0.210	17.56 (0.53, 576.42), *p* = 0.108
IL-8	1.03 (0.18, 5.91), *p* = 0.970	0.57 (0.08, 4.07), *p* = 0.577
IL-12p70	4.65 (0.77, 28.25), *p* = 0.095	13.24 (1.22, 143.27), *p* = 0.033
FGF-2	1.13 (0.08, 17.11), *p* = 0.928	16.24 (0.4, 652.76), *p* = 0.139

Abbreviations: NFT = neurofibrillary tangles. Inflammatory markers were log-10 transformed. Model I was adjusted for age, gender and presence of Moderate/Severe neuritic plaque, while Model II was adjusted for age, gender, presence of Moderate/Severe neuritic plaque and duration of dementia.

Bold fonts indicate significant associations between inflammatory marker and diagnostic group ($p<0.05$, multiple binary regression analysis).

Figure S1. Pro-inflammatory markers in prefrontal and parietal lobes of AD and LBD

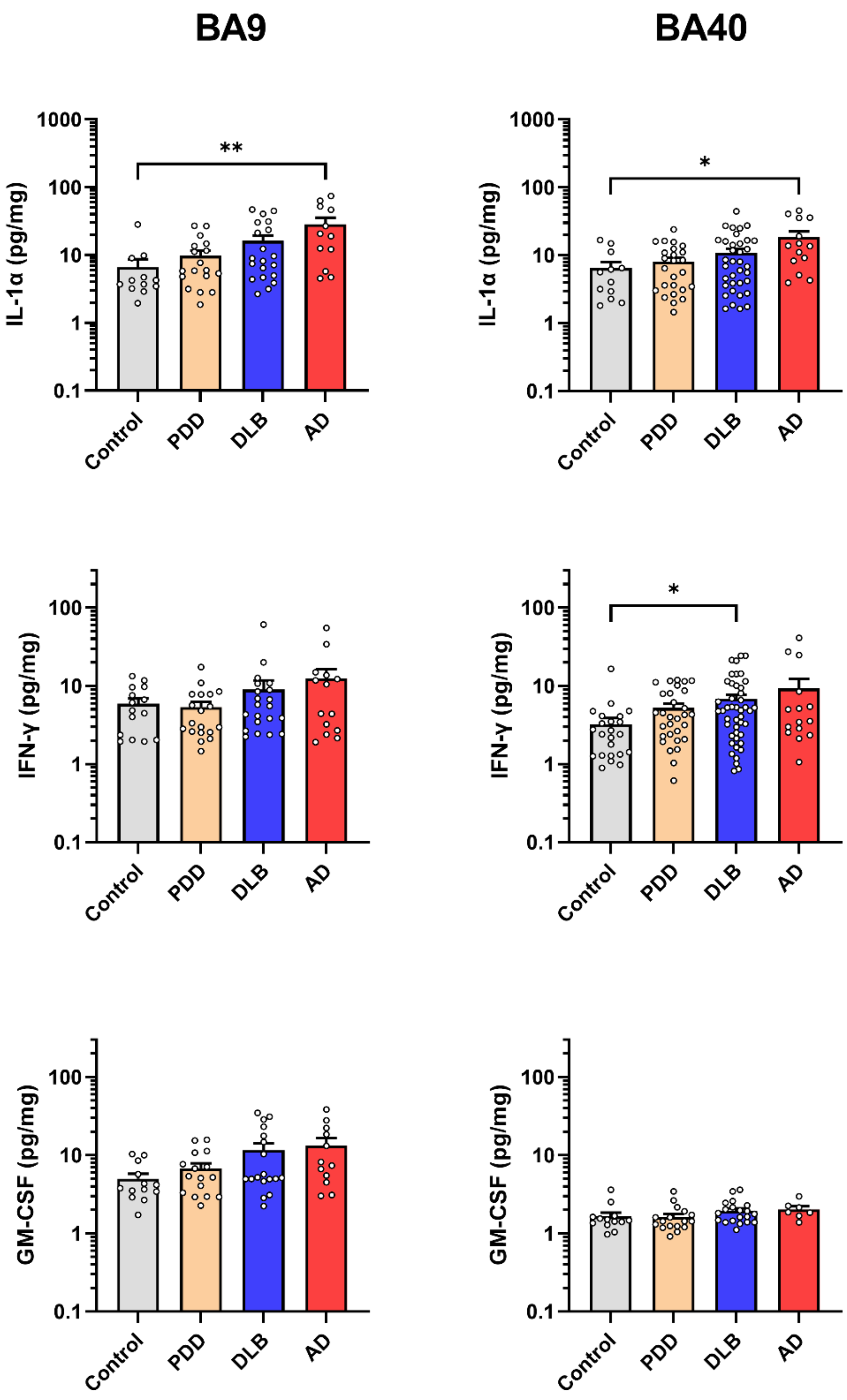

Abbreviations: PDD = Parkinson's disease with dementia, DLB = dementia with Lewy Bodies, AD = Alzheimer's disease. Bar graphs of immunoreactivities (in mean ± SEM, with white dots indicating individual measurements) of each inflammatory markers in prefrontal (BA9) and parietal (BA40) lobes of Control, PDD, DLB and AD patients.

**p* < 0.05 and ***p* < 0.01 indicate significant differences between diagnostic groups (Dunn-Bonferroni *post hoc* tests correction following a significant Kruskal Wallis ANOVA).

Figure S2. Anti-inflammatory markers in frontal and parietal lobes of AD and LBD

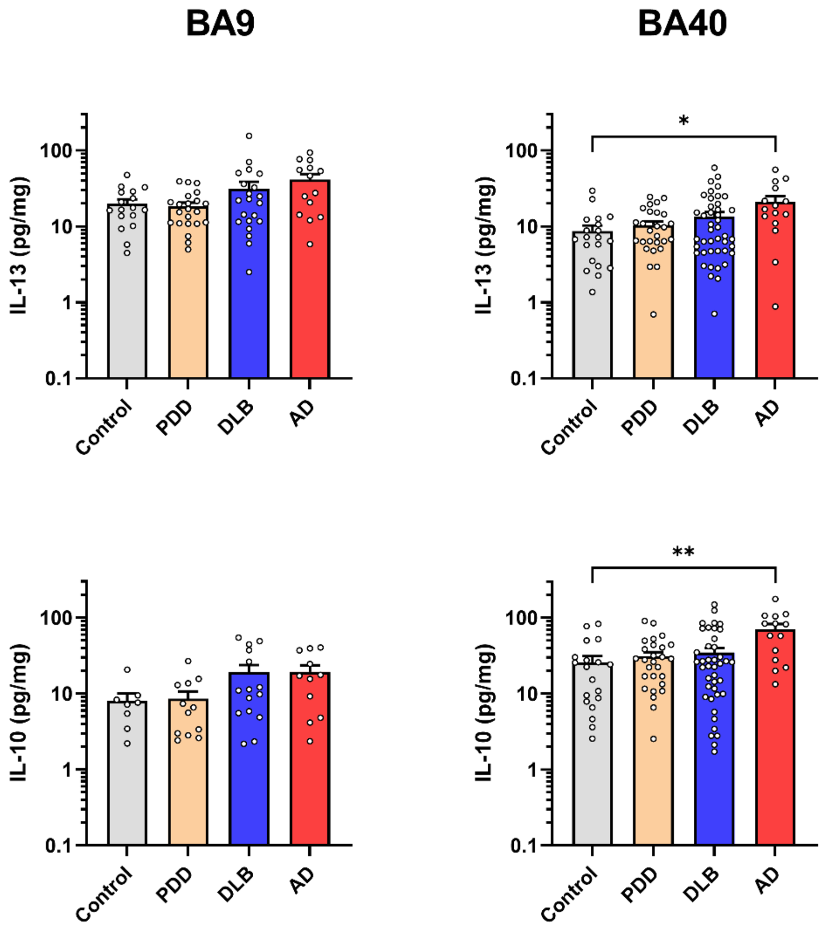

Bar graphs of immunoreactivities (in mean ± SEM, with white dots indicating individual measurements) of each inflammatory markers in prefrontal (BA9) and parietal (BA40) lobes of Control, PDD, DLB and AD patients.

*p < 0.05 and **p < 0.01 indicate significant differences between diagnostic groups (Dunn-Bonferroni *post hoc* tests correction following a significant Kruskal Wallis ANOVA).
